# Child maltreatment and psycho-social impairments among child laborers in rural Bangladesh

**DOI:** 10.1007/s00127-024-02671-9

**Published:** 2024-04-29

**Authors:** Md Abdul Ahad, Yvonne Karen Parry, Eileen Willis, Shahid Ullah

**Affiliations:** 1 Department of Rural Sociology and Development, Sylhet Agricultural University, Sylhet, 3100 Bangladesh; 2 College of Nursing and Health Sciences, Flinders University, SA, Australia; 3 College of Medicine and Public Health, Flinders University, SA, Australia; 4 Research Training Lead, School of Graduate Research, Central Queensland University, Queensland, Australia

**Keywords:** Child maltreatment, Child labor, Psycho-social, Bangladesh

## Abstract

**Purpose:**

The current study focused on exploring the impact of maltreatment of child laborers on their psychosocial health condition from the views of their parents.

**Methods:**

A total of 100 parents of child laborers were recruited using snowball sampling. The structured questionnaire comprised two validated scales including ISPCAN Child Abuse Screening Tool (ICAST-P), and Paediatric Symptom Checklist (PSC) were used for the survey. Factor analysis and multivariable linear regression analysis were performed to examine the data using SPSS version 26, and Stata version 16.1.

**Results:**

A three-factor model consisting of internalizing, externalizing, and attention associated psycho-social impairments of child laborers were derived from the 35-item scale of PSC tool and represented a good fit to the data. A mean estimate of maltreatment indicates that a majority of child laborers are maltreated psychologically, followed by physical maltreatment and neglect. The factor analysis resulted that maltreated child laborers are highly prone to exhibit internalized psycho-social difficulties, followed by externalized and attention-associated emotional and behavioral difficulties among child laborers. The regression model further depicts that child laborers, who had been physically and psychologically maltreated, are significantly more likely to be affected by internalized and attention-related psycho-social impairments.

**Conclusions:**

The study concluded that victimized child laborers exhibited significant internalized, as well as attention-related problems. These findings may be useful for future studies that examine emotional and behavioral problems among maltreated child laborers and, therefore, for developing prevention strategies.

**Supplementary Information:**

The online version contains supplementary material available at 10.1007/s00127-024-02671-9.

## Background

Child maltreatment involves physical maltreatment, psychological and sexual abuse and negelct inflicted upon children. The World Health Organization (WHO) estimated that globally one out of two children aged 2–17 years experiences maltreatment each year [1]. A meta-analysis on child maltreatment also indicates a lifetime prevalence of 22.6% and 36.3% worldwide for physical and psychological maltreatment respectively [2]. It is evident that any forms of maltreatment adversely affect the child’s health, rights and development, which has long been considered an international public health issue [3, 4]. In particular, childhood maltreatment is observed as associated with increased emotional and behavioral difficulties in children compared to physical health affect [5–7]. A meta-analysis estimated that worldwide, 46% of maltreated children are suffering from chronic depression, and over 57% of population with bipolar disorder revealed that they have experienced maltreatment during their childhoods [8]. A plethora of studies identified that victimized children, either physically, psychologically or neglected, extensively suffer from anxiety, sleeping and eating disorders, difficulties concentrating, withdrawal symptoms, antisocial and borderline personality disorders, and suicidal symptoms [6, 9–14]. These adverse outcomes persist from childhood [7] and can lead to severe psychosocial difficulties in long run [15, 16]. Notably, among the different groups of children, child laborers have seen as highly susceptible to be subjected to maltreatment [17].

Child labor itself considered as a serious global public health and social concern. Currently 152 million children are engaged in labor with 70 million working in hazardous conditions [18]. Evidently, in addition to occupational physical injuries caused by child labor, there is also evidence that child laborers are more likely to experience emotional difficulties than children who do not participate in labor [19, 20]. There is evidence that children who are employed are more likely to suffer from internalized psychosocial impairments such as social isolation, hopelessness, low self-esteem, decreased coping efficacy, and externalized psycho-somatic problems including aggressive behavior, a hostile attitude, and academic failure [10, 21–23].

The extent of maltreatment of child laborers is widespread. According to a study exploring the extent of child abuse among working children in Turkey, approximately 62% of child worker apprentices have been abused at work [24]. A number of rigorous studies conducted in the South Asian region have concluded that child labor abuse is endemic on the continent [25–27]. Approximately 46.6% and 40.77% of child laborers in Nepal were found to be victims of intentional physical and psychological abuse, respectively [26], while over 83% were found to be victimized in India [27]. Child laborers in Bangladesh are not free from this curse, as Hadi (2000) noted a substantial proportion of working children are routinely maltreated. Of note, their abusive experiences both at workplaces and home further escalate the risk of being socially and psychologically impaired [17, 26]. This study focused in particular on this complex issue.

In spite of these high spikes of violence against child laborers, very few studies have attempted to assess the impact of maltreatment on the mental health impact of these children [16, 17, 26, 27]. Given that it is already established that child labor has a negative effect on child’s emotional health, the impact is further compounded by violence they endure while in the workplace. Studies on the maltreatment of child laborers have also documented an intensified emotional and behavioral impairments [26–29]. The early detection and treatment of any psychological impairment of children is a primary prevention strategy in any country. However, the emotional well-being of victimized children tends to be neglected in many developing countries, such as Bangladesh, and continues to be ignored by low socio-economic families [30]. Therefore, there is a need, therefore, to gain a comprehensive understanding of the impact of maltreatment of child laborers on their emotional and behavioural well-being.

The purpose of this study is to examine the impact of maltreatment on psycho-social difficulties among child laborers from the perspective of their parents. In particular, this study aimed (i) to identify the psycho-social factor structure using the Paediatric Symptom Checklist (PSC) for child laborers, and (ii) to assess the relationship between maltreatment experiences and psycho-social impairments among child labourers.

## Methods and materials

### Study setting

This cross-sectional study has been conducted in the three Upazilas (sub-district) namely Bishwambarpur, Dharmapasha and Doarabazar of Sunamganj district of Bangladesh. The purposive selection of the three Upazilas was also based on the criterion such as higher illiteracy, poverty rate and agricultural holdings compared with other Upazilas of this district [31, 32].

### Participants

A total of 100 parents of child laborers, aged 10 to 17 years, were selected as a representative population using a snowball sampling method. Snowball sampling were followed since the participants are marginalised group of population, and it would be difficult to reach them randomly [33]. As the parents were asked about the maltreatment history of their children as laborers, the sample size of parents was estimated based on the proportion of child maltreatment (82.4%) in Bangladesh found in a prior study [34], 95% confidence interval and an absolute precision of 7% as per the method proposed by Lwanga & Lemeshow [35]. This resulted in 114 parents with an expected dropout rate of 20%. Later, the study was able to recruit 100 parents, with an 88% response rate.

### Data collection tools

#### ISPCAN child abuse Screening Tool- parent version (ICAST-P)

The ICAST-P questionnaire was adopted to measure the extent of physical, psychological maltreatment and neglect. It measures disciplinary maltreatment practices directed at children [36]. The ICAST-P was found adequate to assess child maltreatment at work and at home [37]. This tool is found validated and reliable (Cronbach alpha: 0.60–0.87) in cultural contexts of South Asian countries [38, 39]. Children’s maltreatment experiences were measured by sixteen physical maltreatment items, eight psychological items, and three neglect items. Items of both physical and psychological maltreatment included six response categories for the past year prevalence of child maltreatment including never, yes but not in the past year, once or twice, 3 to 5 times, 6 to 10 times, and ≥ 10 times and weighted values were 1, 2, 3, 4, 5, and 6 respectively. A mean score was calculated for each form of maltreatment. The mean score ranged from between 0 and 6 for each module of maltreatment [36].

#### Paediatric symptom checklist (PSC)

Typically, standardized instruments are used to estimate psycho-social problems among victimized children worldwide. Mental health professionals use a number of clinically validated tools for screening and diagnosing psychological disorders. However, most of the existing measurement tools are not conducive to apply in all paediatric settings as these are time-consuming, lack standardized norms, require professional training to administer [14, 40]. Additionally, the younger children may not possess sufficient cognitive understanding to comprehend some psycho-matric items [14]. In contrast to these measurement tools, PSC is found as widely used screening inventory, which is easy to administer as only 5 min take to complete scoring and is simple to interpret [40–42]. It provides opportunity to capture the parents’ impressions of their children’s psycho-social conditions [40]. It has been validated for use in clinical, educational, and public health, low-income and minority settings [40, 43]. This tool has been identified as a reliable screening measure (Cronbach alpha: 0.86) for assessing children’s psychosocial problems [44]. In this study, the PSC tool captured the parents’ recognition of the cognitive, emotional and behavioral problems associated with child maltreatment. A total of 35 items were listed in PSC with response items weighted as 0, 1 and 2 for Never, Sometimes and Often [45]. The range of possible scores is between 0 and 70 points, which were calculated by adding the individual values for each item. The threshold score of 28 or above indicates psychological impairment in child laborers [42].

For comparison, the English questionnaire, which included both the ICAST-P and PSC (Parents’ version) tools, was translated into Bangla and back-translated. In the first stage, three academics with expertise in socio-epidemiology and health science research, including one paediatrician with a doctorate and one biostatistician with a doctorate, as well as one registered nurse with a doctorate, translated the English versions of the ICAST tools and PSC tools into Bangla.

Two academics were knowledgeable about the scales, their terminologies, as well as the theoretical construction of the questionnaire. One academic (academic and paediatrician) was familiar with child health related terminologies. He knew colloquial phrases, idioms, and idiomatic expressions in English. In collaboration with the academic experts, we translated the original English version of questionnaires into Bangla.

Two additional academic experts (who were also registered nurses with PhD degrees and experienced in scale validation) reviewed the transcription process with the authors of the study and resolved concerns. In a consensus meeting composed of all the experts (translators) and authors of the study, we analysed and discussed discrepancies in words, meaning, or phrases, and resolved them. As a result, we were able to develop Bangla versions of all questionnaires. Additionally, the Bengali version of the questionnaire was tested on three parents of child laborers to determine whether it was understandable for parents with low literacy levels. We found the translated versions were comprehensible.

In the second stage, an academic expert (Biostatistician), whose native language is Bangla and English is a second language, and one registered nurse with a PhD degree, whose first language is English, but who can also speak Bangla performed a back-translation of the translated Bangla scales into English. Both translators were experienced in international translation. These translated versions were combined to produce one single translated version for each scale. Additionally, we consulted with an academic with expertise in child protection research, whose native language is English and Bangla is a second language, in order to compare and evaluate the translated versions in terms of similarities and conceptual equivalence compared to the original English version and the first Bangla translation. As a result, the Bangla versions of the scales were back translated into English.

### Collection of data

The study conducted face to face interviews with parents of child laborers aged 10 to 17 years. To conduct interviews, the study recruited three skilled investigators, one of who had experience of collecting clinical data. Considering the scale items in mind, they were given a three-day training session to familiar with on the subject matter of the study, building rapport, obtaining consent, maintaining confidentiality, avoiding conflict, and responding appropriately. Data were collected in between April to June 2021.

### Data analysis

Prior to exploring the relationship between ICAST-P and PSC, the study conducted the exploratory factor analysis (EFA) and Confirmatory factor analysis to compress the eligible items of PSC into a small number of factors [46]. The application of EFA is identified as appropriate to identify factor structure of psycho-social impairment among vulnerable children [47].

The study measured Kaiser-Meyer-Olkin (KMO) and the Bartlett’s tests of sphericity to consider whether principal component analysis is appropriate in EFA [46]. The study considered a fixed number of factors instead of considering eigenvalues. Evidently, studies on the factor analysis of PSC reported by parents of children retained a three-factor structure including internalising, externalising and attention factors [41, 48]. Therefore, a varimax rotation approach is applied constrained to these three factors [46]. A loadings value of greater than 0.30 is considered in order to evaluate strong factor loadings [40, 48]. Later, the mean value of each latent constructs with retained PSC items were estimated.

Further, a CFA was conducted to verify the validity of the latent constructs that were derived from the EFA. To assess whether the data are accurately fit to the model developed in EFA, PCMIN/df, Root mean square error of approximation (RMSEA), Comparative fit index (CFI) and PCFI were taken into account. PCMIN/df > 3.0, RMSEA > 0.08, PCFI > 0.5 and CFI > 0.9 are the standard criteria for determining model fitness [49]. The multivariable linear regression analysis was employed to assess the relationship between forms of maltreatment and emerged psycho-social factors (internalizing factors, externalizing factors and attention). A 95% confidence level and significance level of less than 0.05 were set as criteria for determining significant relationship. The overall analysis was performed using SPSS version 26, Stata version 16.1 and R version 4.2.1.

### Ethical approval

To conduct the study and interview with parents of child labourers aged 10 to 17 years, a formal ethical approval was obtained from the Human Research Ethics Committee of the University where the research is being conducted. Additionally, authorization to conduct interviews were also taken from a University of Bangladesh.

Data collectors shared the study description and purpose of the research to parents and ensured that they provided verbal consent to be interviewed. It was explained to them that they could withdraw from giving an answer during the interview session if they feel uncomfortable or reluctant to continue. Participants were assured of confidentiality and compensated $2.5AUD (157 Taka) for their participation in the survey. Researchers further assured them that if they became physically or psychologically ill during the interview session, they would assist them in obtaining medical treatment. Nevertheless, no participants found suffered from any health problems during interviews. In view of the fact that many of them may not understand the questions, the data collector filled in the form on their behalf.

## Results

### Construct validity of PSC tool using EFA and CFA

The fixed three-factor solution resulted in 36.6% of the total variance being explained in the PSC-35 items. In the rotated factor pattern, the estimated factor loadings for items related to psycho-social difficulties were above 0.4, as shown in Table 1.

**Table 1:** Factor loadings for the paediatric symptom checklist (PSC)

PSC items	Internalizing factors	Externalizing factors	Attention	Cronbach alpha
26. Gets hurt frequently	0.65			0.81
12. Is irritable, angry	0.64			
21. Have trouble sleeping	0.62			
22. Worry a lot	0.58			
11. Feels sad, unhappy	0.56			
25. Takes unnecessary risks	0.49			
10. Is afraid of new situations	0.48			
19. Is down on him or herself	0.45			
28. Acts younger than children his or her age	0.43			
13. Feels hopeless	0.42			
34. Takes things that do not belong to him or her		0.63		0.63
31. Does not understand other people’s feelings		0.58		
32. Teases others		0.53		
2. Spends more time alone		0.48		
33. Blames others for his or her troubles		0.47		
35. Refuses to share		0.46		
29. Do not listen to rules		0.43		
15. Less interested in friends		0.41		
17. Absent from school			0.75	0.71
16. Fights with other children			0.74	
18. School grades dropping			0.69	
14. Has trouble concentrating			0.46	

The EFA resulted that Factor 1 (internalizing subscale) is consisted of a total of ten items related to internalized psycho-social symptoms among child laborers. Factor 2 is an externalized psycho-social subscale which combined a total of eight items related to the externalized psycho-social problems of child laborers. The third identified factor is termed as attention subscale which consisted of four attention-oriented psycho-social difficulty items. The measured Cronbach alpha demonstrate that internalizing items are highly reliable (α = 0.81) followed by moderately consistent attention subscale (α = 0.71), and externalizing factor (α = 0.63). Based on CFA, the three-factor model appears adequately fitted to the data, as most fit criteria are acceptable. The Bartlett’s test of sphericity (χ2 = 2390.8, *p* < 0.001) identified the eligibility of conducting a principal component analysis and KMO of 0.6 indicated an adequate sample size for exploratory factor analysis.

### Psycho-social impairments and prevalence of maltreatment of child laborers

The overall mean score of the PSC was 16.4. Specifically, 43% of child laborers exhibited an elevated score (28 or above) on PSC, which indicates a startling episode of psycho-social problems among child laborers. The results in Table 2 indicate the mean of psycho-social factors. This apparently reflect that the victimized child laborers were highly susceptible to exhibit internalized psycho-social symptoms followed by externalized psycho-social symptoms and attention difficulties.

**Table 2:** Descriptive statistics of the identified psycho-social factors

Factors	Mean	SD (range)
Internalizing	7.61	3.91 (0–18)
Externalizing	5.52	2.99 (0–13)
Attention	3.25	2.26 (0–8)

The estimated prevalence of physical, psychological maltreatment and neglect of child laborers from the items of ICAST-P is presented in Tables 4, 5 and 6 (included in Appendices). The prevalence estimation indicates that child laborers are highly susceptible to psychological maltreatment (2.8) followed by physical maltreatment (2.6), and neglect (1.8).

### Multivariable regression analysis of psycho-social factors and child maltreatment reported by parents of child laborers

Data reported by parents indicate that child laborers who have been victimized were significantly screened for internalized and attention-related psychosocial problems, presented in Table 3.

**Table 3:** Multivariate regression analysis of the psycho-social impairments and maltreatment of child laborers

Maltreatment forms	Internalizing factors			Externalizing factors			Attention		
	β	95% CI	*p*	β	95% CI	*p*	β	95% CI	*p*
Physical maltreatment	0.12	0.03, 0.21	0.01	0.03	-0.07, 0.12	0.57	0.22	0.09, 0.36	< 0.01
Psychological maltreatment	0.11	0.01, 0.19	0.02	0.02	-0.06, 0.12	0.56	0.27	0.14, 0.41	< 0.001
Neglect	0.02	-0.25, 0.29	0.89	-0.13	-0.38, 0.13	0.33	0.29	-0.11, 0.68	0.15

**Note**: *β*- Unstandardized beta co-efficient, *p*- *P* value, significant at *p* < 0.05, *p* < 0.01, *p* < 0.001

On adjustment of factors in the multivariable modelling, the study found that child laborers who have experienced physical maltreatment are more likely to be screened for internalized psychosocial symptoms by 0.12 units (*β* = 0.12, 95% CI = 0.03, 0.21, *p* = 0.01). The Table 3 further provides evidence that child laborers were more likely to be screened for internalized psycho-social difficulties by 0.11 units when they were psychologically victimized (β= 0.11, 95% CI = 0.01, 0.19, *p* = 0.02). Of note, no particular type of maltreatment was found to have had a significant impact on child laborers’ externalized psychosocial difficulties. Neglect is also observed as not being significantly related to psychosocial problems experienced by child laborers. In addition, as illustrated in Table 3, child laborers who have experienced physical maltreatment displayed an increase of 0.22 units (*β* = 0.22, 95% CI = 0.09, 0.36, *p* < 0.01) in attention-related psychosocial difficulties. Additionally, psychologically maltreated child laborers were more likely to be screened for attention-related psychosocial difficulties by 0.27 units (*β* = 0.27, 95% CI = 0.14, 0.42, *p* < 0.001), regardless of other forms of maltreatment.

## Discussion

It has been documented that child maltreatment has serious negative effects on the psychological health of children. Among the vicimtized children, socially and economically vulnerable groups of children are highly susceptible to expereicne a variety of psycho-traumatic symptoms [19, 21, 27, 50]. In spite of the fact, the impact of maltreatment of children as laborers remains under-researched [20]. This study attempted to survey the children’spsycho-social difficulties utilizing parents version of PSC and investigate the relationships between psycho-social factors and different forms of maltreatment.

In this study, we found that child laborers, as a vulnerable group of children, show psychosocial impairments associated with internalizing, externalizing, and attention-oriented symptoms, which are also typical psychosocial symptoms of acutely ill children. The retained items derived from factor analysis, under these three psycho-social factors were maintained concordance to the three factors models developed for children with acute illnesses in earlier studies [51–53]. The items included in internalized factors apparently depicting depression, anxiety, withdrawal behavior and other internal psycho-somatic symptoms. Externalizing items reflect aggressive, destructive behavior or conduct disorders, while items in the attention subscale apparently implying the effects of cognitive difficulties among child laborers [40, 48, 54].

The estimate of the parents’ reported data revealed that the victimized children, as laborers, are highly prone to experiencing psychosocial difficulties compared with experiences of different disadvantaged and physically challenged groups of children observed in prior studies [40, 41, 47, 48]. The extent of psycho-social difficulties indicates the need for additional research to determine the long-term psycho-social impacts in the child labor population. Specifically, the study resulted that the victimized child laborers are largely exhibiting internalized psycho-social symptoms followed by externalized problems and attention deficit disorders which were parallelly observed in earlier studies [41, 47, 48]. Similar to the findings of current study, Badrand colleagues found that nearly half of all children who are maltreated exhibit internalized symptoms; depression, stress, and anxiety [54]. These internalized symptoms are highly responsible to disrupt normal brain development in early childhood [54–56]. On the flip side, a study outlined that externalized psycho-social problems and attention difficulties outweigh internalized problems among victims [6].

Apart from an overall impact of child labor maltreatment, the study specifically measured the psycho-social consequences of each type of maltreatment. Consistent with previous studies, the multivariable regression model further identified that child laborers who have been subjected to physical and psychological maltreatment exhibit greater internalizing symptomatology and attention deficit symptoms [15, 56–58]. Similar to the prior studies on child labor maltreatment, the current study also noticed that child laborers who are physically victimized are more prone to exhibit internal emotional difficulties, and psychological maltreatment explicitly resulted in internalized and attention specific psychosocial difficulties [26, 27]. Furthermore, while the current study estimated an insignificant relationship between all forms of maltreatment and externalized psycho-social symptoms, the prior studies identified that maltreated children are more likely to encounter externalized defiant disorders [13, 27, 56]. Child neglect has also been categorized as a potential predicator of psycho-social symptoms. There is ample evidence that neglected children have elevated rates of internalized symptoms (i.e., major depression) and attention difficulties (i.e., ADHD) [56, 59]. However, this study, based on the reports of parents of child laborers observed an insignificant relationship between neglect and psycho-social difficulties among child laborers.

The findings indicate that exposure to physical or psychological maltreatment are significantly more likely to develop internalized and attention-related disorders among child laborers. Therefore, efforts should be made to eliminate all forms of psychological bullying as well as corporal punishment from all settings, although in Bangladesh they are only enforced in educational environments [60]. Specifically, this study shows that victimized child laborers are most likely to experience internalized psychosocial problems, which are often difficult to identify. Therefore, the importance of early detection of psychosocial impairments cannot be overstated. A comprehensive system of early intervention and prevention should be offered by public child protection services in Bangladesh. Additionally, child welfare workers may be able to assist in the assessment of psychosocial symptoms associated with maltreatment, as well as providing information on prevention strategies. Furthermore, this study draws attention to the urgent need for further research into the relationship between child laborers’ experiences of maltreatment and the potential risks of psychosocial difficulties.

### Limitation

The study had a number of limitations. The PSC tools were mostly analysed in the context of physically vulnerable children, whereas this study focused on the maltreatment of child laborers drawing on the views of parents of these children. This resulted in a constraint in bridging the psycho-social difficulties of children in general and those of victims of child labor in particular. Furthermore, the data were collected during the Covid-19 pandemic, limiting the population size compared to prior studies of psycho-social impairments in children.

## Conclusion

Despite of limitations, the study clearly identified that the maltreated child laborers primarily exhibit internalized, externalized and attention related psycho-social problems. The mental health of children engaged in labor, particularly those who have been physically and psychologically maltreated is compromised. It is therefore necessary to conduct further surveys of the psycho-social problems of these vulnerable groups. Ideally, the findings of this study will provide prospective social science and clinical researchers with insight into the psychological health of children in labor.

## Electronic supplementary material

Below is the link to the electronic supplementary material.

Supplementary Material 1

## References

[1] WHO (2020) Global status report on preventing violence against children. World Health Organization: Geneva

[2] Stoltenborgh M et al (2015) The prevalence of child maltreatment across the Globe: review of a series of Meta-analyses. Child Abuse Rev 24(1):37–50

[3] Kim H et al (2017) Lifetime prevalence of investigating child maltreatment among US children. Am J Public Health 107(2):274–280 27997240 10.2105/AJPH.2016.303545 PMC5227926

[4] Moody G et al (2018) Establishing the international prevalence of self-reported child maltreatment: a systematic review by maltreatment type and gender. BMC Public Health 18(1):1164 30305071 10.1186/s12889-018-6044-y PMC6180456

[5] Ahn YD et al (2022) Psychological aspects of child maltreatment. J Korean Neurosurg Soc 65(3):408–414 35508958 10.3340/jkns.2021.0300 PMC9082119

[6] Rodgers CS et al (2004) The impact of individual forms of childhood maltreatment on health behavior. Child Abuse Negl 28(5):575–586 15159071 10.1016/j.chiabu.2004.01.002

[7] Gilbert R et al (2009) Burden and consequences of child maltreatment in high-income countries. Lancet 373(9657):68–81 19056114 10.1016/S0140-6736(08)61706-7

[8] Post RM et al (2013) More stressors prior to and during the course of bipolar illness in patients from the United States compared with the Netherlands and Germany. Psychiatry Res 210(3):880–886 24021999 10.1016/j.psychres.2013.08.007

[9] Edwards VJ et al (2003) Relationship between multiple forms of childhood maltreatment and adult mental health in community respondents: results from the adverse childhood experiences study. Am J Psychiatry 160(8):1453–1460 12900308 10.1176/appi.ajp.160.8.1453

[10] Ryan KD et al (2000) Psychological consequences of child maltreatment in homeless adolescents: untangling the unique effects of maltreatment and family environment, vol 24. Child Abuse & Neglect, pp 333–352. 3 10.1016/s0145-2134(99)00156-8 10739077

[11] Kaplan SJ et al (1998) Adolescent physical abuse: risk for adolescent psychiatric disorders. Am J Psychiatry 155(7):954–959 9659863 10.1176/ajp.155.7.954

[12] Ahad M et al (2021) Child abuse during the covid-19 pandemic - prospect, risk and factors: a narrative review. Hellenic J Psychol 18:46–62

[13] Liu RT (2019) Childhood maltreatment and impulsivity: a Meta-analysis and recommendations for future study. J Abnorm Child Psychol 47(2):221–243 29845580 10.1007/s10802-018-0445-3 PMC6269232

[14] Briere J et al (2001) The Trauma Symptom Checklist for Young Children (TSCYC): reliability and association with abuse exposure in a multi-site study, vol 25. Child Abuse & Neglect, pp 1001–1014. 8 10.1016/s0145-2134(01)00253-8 11601594

[15] Celik SSPRN, Baybuga MSPRN (2009) Verbal, physical and sexual abuse among children working on the street. Australian J Adv Nurs (Online) 26(4):14–22

[16] Gharaibeh M, Hoeman S (2003) Health hazards and risks for abuse among child labor in Jordan. J Pediatr Nurs 18(2):140–147 12720212 10.1053/jpdn.2003.31

[17] Das R, Chen A (2019) Towards a theoretical Framework for understanding Capitalist Violence against Child Labor. World Rev Political Econ 10(2):191–219

[18] Ahad M, Parry Y, Willis E (2020) Spillover trends of Child Labor during the Coronavirus Crisis- an Unnoticed Wake-Up Call. Front Public Health 8:488 33014979 10.3389/fpubh.2020.00488 PMC7500159

[19] Al-Gamal E et al (2013) The psychosocial impact of child labour in Jordan: a national study. Int J Psychol 48(6):1156–1164 23586366 10.1080/00207594.2013.780657

[20] Jayawardana D, Baryshnikova NV, Cheng TC (2022) The long shadow of child labour on adolescent mental health: a quantile approach. Empirical Economics, : p. 1–21

[21] Sturrock S, Hodes M (2016) Child labour in low- and middle-income countries and its consequences for mental health: a systematic literature review of epidemiologic studies. Eur Child Adolesc Psychiatry 25(12):1273–1286 27217156 10.1007/s00787-016-0864-z

[22] Hesketh TM et al (2012) The psychosocial impact of child domestic work: a study from India and the Philippines. Arch Dis Child 97(9):773–778 22685048 10.1136/archdischild-2012-301816

[23] Nuwayhid IA et al (2005) Health of children working in small urban industrial shops. Occup Environ Med 62(2):86 15657189 10.1136/oem.2004.015503 PMC1740960

[24] Öncü E et al (2013) Abuse of working children and influencing factors, Turkey. Child Abuse & Neglect, p 37 10.1016/j.chiabu.2012.11.006 23312120

[25] Hadi A (2000) Child abuse among working children in rural Bangladesh: prevalence and determinants. Public Health 114(5):380–384 11035460 10.1038/sj.ph.1900664

[26] Dhakal S et al (2019) History of abuse and neglect and their associations with mental health in rescued child labourers in Nepal. Aust N Z J Psychiatry 53(12):1199–1207 31185738 10.1177/0004867419853882

[27] Pandey R et al (2020) Childhood maltreatment and its mental health consequences among Indian adolescents with a history of child work. Aust N Z J Psychiatry 54(5):496–508 32156147 10.1177/0004867420909524 PMC7227131

[28] Celik S, The (2009) Verbal Phys Sex Abuse among Child Working Str 26(4):14

[29] Kiss L et al (2015) Exploitation, Violence, and suicide risk among child and adolescent survivors of human trafficking in the Greater Mekong Subregion. JAMA Pediatr 169(9):e152278–e152278 26348864 10.1001/jamapediatrics.2015.2278

[30] Prince M et al (2007) No health without mental health. Lancet 370(9590):859–877 17804063 10.1016/S0140-6736(07)61238-0

[31] BBS (2013) District statistics 2011 Sunamganj. Ministry of Planning, Government of the People’s Republic of Bangladesh

[32] BBS (2015) Report on Child Labor Survey (CLS), Bangladesh 2013. Statistics and Information Division, Ministry of Planning, Bangladesh

[33] Tenzek KE (2017) The SAGE Encyclopedia of Communication Research methods. SAGE Publications, Inc, Thousand Oaks, California

[34] BBS (2016) and UNICEF, *Child well-being survey in urban areas of Bangladesh, key results*

[35] Lwanga SK, Lemeshow S (1991) Sample size determination in health studies: a practical manual. World Health Organization, Geneva

[36] Runyan DK et al (2009) The development and piloting of the ISPCAN child abuse Screening Tool—parent version (ICAST-P). Child Abuse Negl 33(11):826–832 19854511 10.1016/j.chiabu.2009.09.006

[37] O’Leary P et al (2018) Violence against children in Afghanistan: concerns and opportunities for positive change. Child Abuse Negl 76:95–105 29096162 10.1016/j.chiabu.2017.10.010

[38] Chen C et al (2020) Psychometric testing of the Chinese version of ISPCAN child abuse screening tools parent’s version (ICAST-P). Child Youth Serv Rev 109:104715

[39] Lakhdir MPA et al (2021) Factors Associated with child maltreatment among children aged 11 to 17 years in Community settings of Karachi, Pakistan, using Belsky Ecological Framework. J Interpers Violence 36(1–2):297–313 29294892 10.1177/0886260517726973

[40] Stoppelbein L et al (2005) Factor analysis of the Pediatric Symptom Checklist with a chronically Ill Pediatric Population. J Dev Behav Pediatr 26(5):349–355 16222174 10.1097/00004703-200510000-00002

[41] Reijneveld SA et al (2006) Use of the Pediatric Symptom Checklist for the detection of psychosocial problems in preventive child healthcare. BMC Public Health 6(1):197 16872535 10.1186/1471-2458-6-197 PMC1550396

[42] Jellinek MS et al (1988) Pediatric Symptom Checklist: Screening school-age children for psychosocial dysfunction. J Pediatr 112(2):201–209 3339501 10.1016/s0022-3476(88)80056-8

[43] Murphy JM et al (1996) Utility of the Pediatric Symptom Checklist as a psychosocial screen to meet the federal early and periodic screening, diagnosis, and treatment (EPSDT) standards: a pilot study. J Pediatr 129(6):864–869 8969728 10.1016/s0022-3476(96)70030-6

[44] Anderson DL et al (1999) Use of the pediatric symptom checklist in the pediatric neurology population. Pediatr Neurol 20(2):116–120 10082339 10.1016/s0887-8994(98)00121-0

[45] Jellinek MS et al (1999) Use of the Pediatric Symptom Checklist to screen for psychosocial problems in pediatric primary care: a national feasibility study, vol 153. Archives of pediatrics & adolescent medicine, pp 254–260. 3 10.1001/archpedi.153.3.254 PMC3905751 10086402

[46] Pallant J (2020) SPSS survival manual: a step by step guide to data analysis using IBM SPSS. Routledge, London

[47] Simonian SJ, Tarnowski KJ (2001) Utility of the Pediatric Symptom Checklist for behavioral screening of disadvantaged children. Child Psychiatry Hum Dev 31(4):269–278 11227987 10.1023/a:1010213221811

[48] Reed-Knight B et al (2011) Factor structure of the pediatric symptom checklist with a pediatric gastroenterology sample. J Clin Psychol Med Settings 18(3):299–306 21512749 10.1007/s10880-011-9242-7

[49] Brown TA, Moore MT *Confirmatory factor analysis* Handbook of structural equation modeling, 2012. 361: p. 379

[50] Zhao J et al (2019) Childhood maltreatment influences mental symptoms: the mediating roles of emotional intelligence and social support. Front Psychiatry 10:415 31316399 10.3389/fpsyt.2019.00415 PMC6611427

[51] Wolfe-Christensen C et al (2014) Factor analysis of the Pediatric Symptom Checklist in a Population of children with voiding dysfunction and/or nocturnal enuresis. J Clin Psychol Med Settings 21(1):72–80 24158241 10.1007/s10880-013-9375-y

[52] Sheldrick RC et al (2012) The Preschool Pediatric Symptom Checklist (PPSC): development and initial validation of a New Social/Emotional Screening Instrument. Acad Pediatr 12(5):456–467 22921494 10.1016/j.acap.2012.06.008 PMC3907071

[53] Ardıç E, Ünsal G, Bayram S (2020) Validity and reliability of the Pictorial Pediatric Symptom Checklist. J Psychiatric Nurs 11(1):20–27

[54] Badr HE et al (2018) Childhood maltreatment: a predictor of mental health problems among adolescents and young adults. Child Abuse Negl 80:161–171 29609135 10.1016/j.chiabu.2018.03.011

[55] Bala G et al (2012) Psychometric properties of the Pediatric Symptom Checklist in preschool children in Serbia. Medicinski Glasnik, 9(2) 22926377

[56] Mills R et al (2013) Child maltreatment and adolescent mental health problems in a large birth cohort. Child Abuse Negl 37(5):292–302 23380430 10.1016/j.chiabu.2012.11.008 PMC3918944

[57] Leeb RT, Lewis T, Zolotor AJ (2011) Rev Phys Mental Health Consequences Child Abuse Negl Implications Pract 5(5):454–468

[58] Ford JD et al (2000) Child maltreatment, other trauma exposure, and posttraumatic symptomatology among children with oppositional defiant and attention deficit hyperactivity disorders. Child Maltreat 5(3):205–217 11232267 10.1177/1077559500005003001

[59] Briscoe-Smith AM, Hinshaw SP (2006) Linkages between child abuse and attention-deficit/hyperactivity disorder in girls: behavioral and social correlates. Child Abuse Negl 30(11):1239–1255 17097140 10.1016/j.chiabu.2006.04.008 PMC1934403

[60] Shahidullah SM (2017) *Criminalization of Child Abuse and Violence against Children in South Asia: Law and Legal Advances in India, Pakistan, and Bangladesh*, in *Crime, Criminal Justice, and the Evolving Science of Criminology in South Asia: India, Pakistan, and Bangladesh*, S.M. Shahidullah, Editor. Palgrave Macmillan UK: London. pp. 109–144

