# Supplementary material for: Child maltreatment and psycho-social impairments among child laborers in rural Bangladesh
Source: Soc Psychiatry Psychiatr Epidemiol. 2024 Apr 29;60(3):763–70. doi: 10.1007/s00127-024-02671-9 (PMC11870881; doi:10.1007/s00127-024-02671-9)
Supplement: Supplementary file 1 — Supplementary Material 1 [file 127_2024_2671_MOESM1_ESM.docx]

**Appendix**

**Table 4:** Prevalence of physical maltreatment of child laborers aged 10-17 years

| **Maltreatment Categories** | **>10 times**  **(%)** | **6-10 times (%)** | **3-5 times (%)** | **Once or**  **twice**  **(%)** | **Not in past year**  **(%)** | **Never**  **(%)** |
| --- | --- | --- | --- | --- | --- | --- |
| Shook | - | - | - | 10.7 | 13.1 | 75.0 |
| Hit on the buttocks | 1.1 | 18.0 | 42.7 | 30.3 | - | - |
| Hit elsewhere (not buttocks) | 1.2 | 18.1 | 34.9 | 27.7 | 4.8 | 6.0 |
| Twisted ear | 2.2 | 22.2 | 35.6 | 22.2 | - | 15.6 |
| Hit on head | 1.1 | 12.1 | 22.0 | 35.2 | 3.3 | 15.4 |
| Pulled hair | 2.1 | 11.7 | 39.4 | 22.3 | 13.8 | 10.6 |
| Kicked with a foot | 2.2 | 13.0 | 23.9 | 28.3 | 15.2 | 15.2 |
| Put chili pepper or spicy food in mouth | 1.1 | 2.2 | 11.2 | 11.2 | 4.5 | 69.7 |
| Forced to kneel | - | 5.3 | 20.2 | 31.9 | - | 42.6 |
| Spanked on the bottom | 2.2 | 20.9 | 20.9 | 20.9 | 7.7 | 24.2 |
| Choked or squeezed neck | - | 1.0 | 3.1 | 26.8 | 2.1 | 66.0 |
| Pinched | - | 3.1 | 15.5 | 23.7 | 12.4 | 40.2 |
| Slapped on face or back of head | 4.2 | 13.5 | 26.0 | 20.8 | 8.3 | 26.0 |
| Used a hand or pillow to prevent breathing | 2.1 | 10.6 | - | 18.1 | 7.4 | 60.6 |
| Burned, scalded, or branded | - | 3.2 | 5.3 | 22.3 | 5.3 | 63.8 |
| Hit over and over again | 2.1 | 13.4 | 26.8 | 19.6 | 6.2 | 32.0 |

**Table 5:** Prevalence of psychological maltreatment of child laborers aged 10-17 years

| **Maltreatment Categories** | **>10 times** | **6-10 times** | **3-5 times** | **Once or twice** | **Not in past year** | **Never** |
| --- | --- | --- | --- | --- | --- | --- |
| Threatened to leave or abandon | 4.0 | 18.0 | 30.0 | 17.0 | 3.0 | 28.0 |
| Shouted, yelled, or screamed | 9.2 | 37.8 | 26.5 | 20.4 | 2.0 | 3.1 |
| Threatened to invoke ghosts or evil spirits | 7.2 | 16.5 | 16.5 | 12.4 | 2.1 | 43.3 |
| Cursed | - | - | 21.0 | 22.0 | 8.0 | 40.0 |
| Threatened to kick out of house | 5.2 | 13.4 | 20.6 | 15.5 | 2.1 | 42.3 |
| Locked out of house | 2.0 | 5.0 | 12.0 | 27.0 | 5.0 | 49.0 |
| Insulted by calling names | 11.1 | 32.3 | 22.2 | 14.1 | 8.1 | 12.1 |
| Refused to speak | 2.2 | 12.9 | 24.7 | 20.4 | - | 39.8 |
| Withheld a meal as punishment | 3.1) | 15.3 | 22.4 | 19.4 | 6.1 | 28.6 |
| Locked in a dark room | 1.0 | 1.0 | 7.1 | 19.2 | 3.0 | 65.7 |
| Used public humiliation | - | 8.3 | 20.8 | 19.8 | 4.2 | 43.8 |

**Table 6:** Prevalence of neglect of child laborers aged 10-17 years

| **Neglect** | **Yes (%)** |
| --- | --- |
| Deprived of medical care | 78.0 |
| Didn’t get the food or liquid | 63.0 |
| Child was seriously hurt or injured when you or another adult supervising | 61.0 |
